# Deficiency in NDH-cyclic electron transport retards heat acclimation of photosynthesis in tobacco over day and night shift

**DOI:** 10.3389/fpls.2023.1267191

**Published:** 2023-10-31

**Authors:** You Zhang, Yanfei Fan, Xiaotong Lv, Xiyu Zeng, Qiqi Zhang, Peng Wang

**Affiliations:** ^1^ CAS Center for Excellence in Molecular Plant Sciences, Institute of Plant Physiology and Ecology, Chinese Academy of Sciences, Shanghai, China; ^2^ University of Chinese Academy of Sciences, Beijing, China

**Keywords:** photosynthesis, cyclic electron flow (CEF), NDH complex, thermal acclimation, moderate heat stress

## Abstract

In order to cope with the impact of global warming and frequent extreme weather, thermal acclimation ability is particularly important for plant development and growth, but the mechanism behind is still not fully understood. To investigate the role of NADH dehydrogenase-like complex (NDH) mediated cyclic electron flow (CEF) contributing to heat acclimation, wild type (WT) tobacco (*Nicotiana tabacum*) and its NDH-B or NDH-C, J, K subunits deficient mutants (*ΔB* or *ΔCJK*) were grown at 25/20°C before being shifted to a moderate heat stress environment (35/30°C). The photosynthetic performance of WT and *ndh* mutants could all eventually acclimate to the increased temperature, but the acclimation process of *ndh* mutants took longer. Transcriptome profiles revealed that *ΔB* mutant exhibited distinct photosynthetic-response patterns and stress-response genes compared to WT. Metabolite analysis suggested over-accumulated reducing power and production of more reactive oxygen species in *ΔB* mutant, which were likely associated with the non-parallel recovery of CO_2_ assimilation and light reactions shown in *ΔB* mutant during heat acclimation. Notably, in the warm night periods that could happen in the field, NDH pathway may link to the re-balance of excess reducing power accumulated during daytime. Thus, understanding the diurnal cycle contribution of NDH-mediated CEF for thermal acclimation is expected to facilitate efforts toward enhanced crop fitness and survival under future climates.

## Introduction

Photosynthesis is an important biological process that converts light energy into chemical energy and fixes CO_2_ into carbohydrates. This process that is indispensable for the majority of organisms on Earth is sensitive to temperature (Rashid et al., 2020). As global warming proceeds, plants will experience increasing growth temperature and more frequent extreme heat waves (Jagadish et al., 2021). A meta-analysis based on more than 1700 simulations suggested that the negative influence of the warming climate on crop yields will be significantly severe in the 2030s (Challinor et al., 2014). When growth temperature increases above the optimum range, plants are able to conduct a series of responses to the ambient temperature, which could be divided into three levels: avoidance, acclimation, and protection (Hayes et al., 2021). Under mild heat stress within the physiological range, plants execute a process called thermomorphogenesis that regulates morphology and development to avoid exposure to potential heat damage (Crawford et al., 2012; Casal and Balasubramanian, 2019). Under severe heat, the protection responses aim to resist the breakdown of cellular structures, which could be lethal to plants (Vacca et al., 2004; Hayes et al., 2021). Under sustained moderate heat stress, plants have the ability to adjust to environmental changes, and the process is called acclimation, which leads to physiological optimization of net photosynthetic assimilation rate (A_n_) (Atkin et al., 2006; Campbell et al., 2007; Rashid et al., 2020). However, the understanding of molecular mechanisms of photosynthetic thermal acclimation is still limited. Enhancing the photosynthetic thermal acclimation of cereal crops is of necessity to secure food production to meet the increasing global population required, given the rising global temperature.

Photosynthesis can be divided into two main stages, the light reactions and the Calvin–Benson–Bassham (CBB) cycle. The light reaction occurs with the participation of Photosystem II (PSII), Photosystem I (PSI), and other photosynthetic proteins in the thylakoid membranes. Linear electron transport (LET) refers to the process in which PSII captures light energy, stimulates electron transport through plastoquinone (PQ) pool, cytochrome b_6_/f, to PSI and ultimately to NADP^+^. The LET process leads to the accumulation of NADPH, while also creates a proton (H^+^) gradient that energizes the synthesis of ATP (Haehnel, 1984; Yamori and Shikanai, 2016; Shikanai, 2020). Electrons could be alternatively transferred back to PQ from PSI via ferredoxin (Fd), and then return to PSI forming a cyclic flow of transport. This phenomenon is called cyclic electron flow (CEF), with the function of synthesizing extra ATP without accumulation of NADPH, which could balance the ATP/NADPH ratio. When plants are under abnormal physiological environments, CEF is essential for regulating the photosynthetic processes in response to stress conditions (Shikanai, 2014; Yamori and Shikanai, 2016; Shikanai, 2020). Two distinct CEF pathways were found in angiosperms; one is mediated by PROTON GRADIENT REGULATION 5 (PGR5) and PGR5-like Photosynthetic Phenotype 1 (PGRL1) (Munekage et al., 2002; Dalcorso et al., 2008; Yamori and Shikanai, 2016; Ma et al., 2021); the other is dependent on a chloroplast NADH dehydrogenase-like (NDH) complex (Burrows et al., 1998; Kofer et al., 1998; Shikanai et al., 1998; Horvath et al., 2000; Yamori and Shikanai, 2016). The second stage of photosynthesis, CBB cycle, takes place in the stroma of chloroplast (Bassham et al., 1950). CBB cycle uses ATP and NADPH produced from the light reactions to fix CO_2_ into carbohydrates (Raines, 2003).

The chloroplast NDH complex is located on the thylakoid membrane and consists of 11 chloroplast-encoded subunits (NdhA-NdhK), and at least 18 nucleus-encoded subunits (Shen et al., 2021). The complex was first recognized in *Nicotiana tabacum* (common tobacco) in 1987 (Matsubayashi et al., 1987); and its cryo-electron microscopy structure in higher plants was reported recently (Shen et al., 2021). A transient post-illumination rise (PIR) of chlorophyll (Chl) fluorescence represents NDH-dependent reduction of the PQ pool, and has been widely used as an indicator of cyclic electron flow; however, the absence of a single subunit of the whole NDH complex could result in the absence of PIR (Asada et al., 1993; Gotoh et al., 2010). The mutants of NDH subunits are sensitive to increased temperature, which indicates an important role of NDH-mediated CEF under heat stress (Wang et al., 2006; Yamori et al., 2011; Wang et al., 2020). Studies also recognized the roles of NDH-mediated CEF for photosynthesis and plant growth under many other unfavourable environments, such as high light, fluctuating light, low-temperature and drought (Yamori and Shikanai, 2016).

Cyclic electron transport is strongly associated with the redox state of chloroplast and the production of reactive oxygen species (ROS). Previous studies have proposed that hydrogen peroxide (H_2_O_2_) could induce increased transcript level and protein content of NDH subunits, which should contribute to the activity of NDH-mediated electron transport (Casano et al., 2001; Lascano et al., 2003; Strand et al., 2015). Redox state and ROS metabolism also play important roles in plant acclimation to abiotic stress (Pogson et al., 2008; Suzuki et al., 2012). Not only in chloroplast, but also in mitochondria, the electron transport chains are connected with a steady-state balance of carbon metabolism, via reducing equivalents [NAD(P)H] and energy (ATP) (Couee et al., 2006; Rhoads and Subbaiah, 2007; Foyer and Noctor, 2009). Thermal acclimation of photosynthesis and respiration was reported to be asynchronous in previous studies, which is dependent on the timeframe of temperature increase (Campbell et al., 2007; Rashid et al., 2020). Considering the diurnal variation, it is possible that the acclimation has two modes separately during day and night. Daytime carbon metabolism is mainly driven by photosynthesis, and leaf respiration is low-flux during the day, whereas at night, respiration is more significant (Atkin et al., 1998; Atkin et al., 2000). During the day, reductants are able to be transferred from chloroplast to mitochondria through different pathways, such as malate-oxaloacetate shuttle and triosephosphate-3-phosphoglycerate shuttle (Heineke et al., 1991; Scheibe et al., 2005; Yoshida et al., 2007; Lim et al., 2020). It is still inconclusive whether mitochondrial electron transport chain (mETC) at night could continue resolving the excess-accumulated reducing power from chloroplast (Heineke et al., 1991; Shameer et al., 2019).

In order to better understand the association between cyclic electron transport and plant photosynthetic thermal acclimation, this study investigated the effects of deficient in NDH-mediated CEF on thermal acclimation of net photosynthesis, under 3 days timeframe including day and night shift. Previous studies of RNA sequencing showed a large-scale differential gene expression involving a quantity of biological pathways when plants were under temperature perturbations (Hu et al., 2014; Bhardwaj et al., 2015); nevertheless, the changes in protein abundance might not have the same trend as that in transcript abundance (Armstrong et al., 2008; Sidaway-Lee et al., 2014). To assess the role NDH-mediated CEF plays in thermal acclimation more comprehensively, we conducted multiple analysis at the transcript, protein, metabolism, and physiology levels. *Nicotiana tabacum* (common tobacco) was selected as the experimental material due to its abundant research background in NDH complex and NDH-related mutant germplasm resources. The study is targeted to a) find evidence to show NDH-mediated CEF is associated with photosynthetic thermal acclimation after tobacco plants were exposed to moderate heat treatment; b) clarify the related changes in transcript, protein, and metabolite abundance during the acclimation process; c) understand how chloroplast and mitochondria alternate electron transport to re-achieve redox steady-state.

## Results

### Photosynthetic acclimation to moderate higher temperature is delayed in NDH-deficient mutant

**Figure 1:**
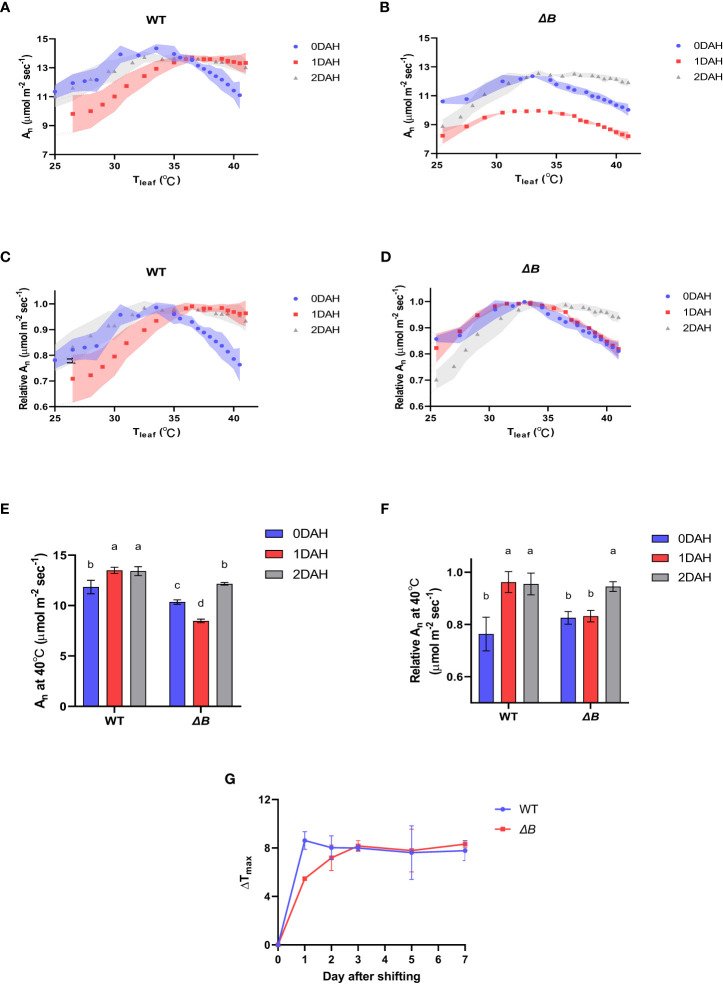
Delayed photosynthetic heat acclimation in tobacco *ndhB* mutant over day and night shift. **(A)** Temperature-response curves of An at different time points after heating in WT; **(B)** temperature-response curves of A_n_ at different time points after heating in *ΔB*; **(C)** temperature-response curves of relative A_n_ at different time points after heating in WT; **(D)** temperature-response curves of relative A_n_ at different time points after heating in *ΔB*; **(E)** A_n_ measured at 40°C at different time points after heating; **(F)** relative A_n_ at 40°C at different time points after heating. A_n_ was measured under 600 µmol photons m^-2^ s^-1^, 400 µmol mol^-1^ CO_2_, and from 25°C to 40°C increasing temperature **(A–D)** or at 40°C **(E, F)**. Relative A_n_ values were normalized to the maximum values of A_n_. Shadow area **(A–D)** or error bars **(E, F)** represent standard deviation of four biological replicates. Different lower-case letters indicate significant (*P* < 0.05) differences by two-way ANOVA **(E, F)**. **(G)** Line plots of T_max_ changes of WT and *ΔB* plants after temperature shifting. Values represent the differences between the T_max_ at corresponding time points and the T_max_ before heating. Error bars represent standard deviation of four biological replicates.

Photosynthetic thermal acclimation is indicated by the shift of net photosynthetic assimilation rate (A_n_) temperature-response curve and the shift of temperature at which maximum A_n_ was achieved (Tmax). When tobacco wild type (WT) and the *ndhB* mutant (*ΔB*, deficient in NDH-B subunit) were grown at normal temperature (25 °C day/20 °C night) and measured with an increasing temperature gradient of 20 °C to 45 °C, the A_n_ of both WT and *ΔB* showed similar temperature-response curve peaked between 30 °C and 35 °C (T_max_ between 30 °C and 35 °C). After the tobacco plants were placed at 35 °C day/30 °C night for 1 day (including night period), the temperature-response curve of WT shifted toward a higher temperature range, with the T_max_ increased to above 35 °C, and the A_n_ decreasing phase became much slower (**Figures 1A, C**). In *ΔB* mutant, the photosynthetic performance was suppressed severely, and without a shift of T_max_ at 1 day after heat-treatment (DAH) (**Figures 1B, D**). The result indicated that compared with the photosynthetic inhibition in the *ndh* mutant at 1DAH, WT plants were rapidly acclimated during the first day in moderate hot environment, and the photosynthesis performance was kept high at above 35°C (**Figures 1A, C**). The shift of temperature-response curve and T_max_ occurred 2 days after heat treatment in *ΔB* mutant, while the shifted curve and T_max_ were retained in WT (**Figures 1G**), indicating both materials achieved heat acclimated status after 2 days treatment.

**Figure 2:**
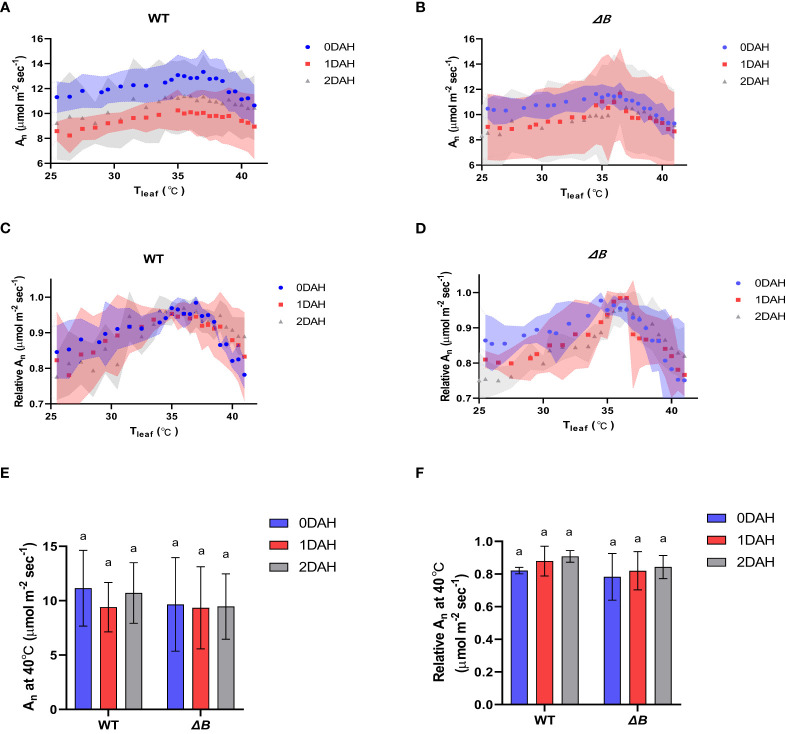
Suppressed photosynthetic heat acclimation in both WT and *ΔB* mutant under continuous light. **(A)** Temperature-response curves of A_n_ at different time points after heating in WT; **(B)** temperature-response curves of A_n_ at different time points after heating in *ΔB*; **(C)** temperature-response curves of relative A_n_ at different time points after heating in WT; **(D)** temperature-response curves of relative A_n_ at different time points after heating in *ΔB*; **(E)** A_n_ measured at 40°C at different time points after heating; **(F)** relative A_n_ at 40°C at different time points after heating. A_n_ was measured under 600 µmol photons m^-2^ s^-1^, 400 µmol mol^-1^ CO_2_, and from 25°C to 40°C increasing temperature **(A–D)** or at 40°C **(E, F)**. Relative A_n_ values were normalized to the maximum values of A_n_. Shadow area **(A-D)** or error bars **(E, F)** represent standard deviation of four biological replicates. Different lower-case letters indicate significant (*P* < 0.05) differences by two-way ANOVA **(E, F)**.

At excessive hot temperature of 40°C, the photosynthesis rate of WT was enhanced at 1DAH and 2DAH (**Figure 1E**). The relative net photosynthesis rates of WT (normalised to maximum A_n_) measured at 40°C 1 day after heat-treatment and 2 DAH, were increased from 70-80% to above 90% of the maximum A_n_ (**Figures 1F**); that is, WT tobacco had enhanced its photosynthesis performance at the high-temperature regime within the 1st day of temperature shift. However, the A_n_ of *ΔB* mutant measured at 40°C was suppressed at 1DAH and did not recover until 2 DAH, which indicates that the acclimation process in *ΔB* mutant was delayed (**Figures 1E, F**). The experiment using another *ndh* mutant, *ΔCJK*, presented a similar trend as *ΔB* compared to WT (**Supplementary Figure 1**). Considering the potential impacts of night period, photosynthesis temperature-response curves were also measured on WT and *ΔB* plants that have been heat-treated under 24 hours full-light. Unlike the rapid shift of WT under 35 °C day/30 °C night within 1 day, the photosynthetic acclimation of WT plants under full-light treatment was slow and indistinct, and was not significantly different from *ΔB* mutant (**Figure 2**).

### Early response of NDH-CEF is linked to heat adaptation of both light reaction and CO_2_ assimilation

**Figure 3:**
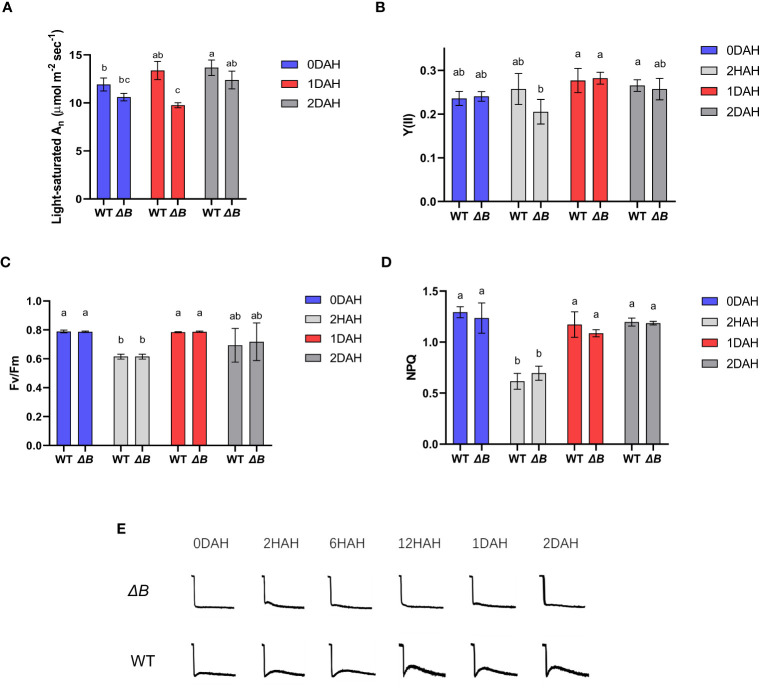
Unparallel acclimation of light reaction and CO_2_ assimilation is associated with heat induced NDH-CEF. Light-saturated A_n_ (under 1000 µmol photons m^-2^ s^-1^) **(A)**, quantum yield in PSII [Y(II)] **(B)**, F_v_/F_m_ **(C)**, and non-photochemical quenching (NPQ) **(D)** of WT and *ΔB* measured at growth temperature and different time points after heat treatment. Error bars represent standard deviation of four biological replicates; different lower-case letters indicate significant (P < 0.05) differences by two-way ANOVA. **(E)** The chlorophyll fluorescence post-illumination rise (PIR) in WT and *ΔB* measured under growth environment at different time points after temperature increase.

To track down the cause and effect of this delayed photosynthetic thermal acclimation, more photosynthetic parameters were gathered at the treatment temperature of 35 °C. Light-saturated A_n_ in WT tobacco was increased after moderate heat treatment; however, the light-saturated A_n_ of *ΔB* was suppressed at 1 DAH, and staged a recovery at 2 DAH (**Figure 3A**). Differently, a decrease of the quantum yield in PSII [Y(II)] was observed earlier at 2 hours after heat treatment (HAH) in *ΔB* compared to WT, although it was recovered and slightly increased in both WT and *ΔB* at 1 DAH and 2 DAH (**Figure 3B**). The different trends between the two photosynthetic parameters in WT and *ΔB* mutant suggested asynchronous responses in light reactions and CBB cycle at 1 DAH. Other chlorophyll fluorescence parameters such as the maximum quantum efficiency of PSII (F_v_/F_m_) and Non-photochemical quenching (NPQ) were all similarly suppressed at the initial period of heating (2 HAH), and recovered at 1 DAH and 2 DAH, in both WT and *ΔB* (**Figures 3C, D**).

Post-illumination rise (PIR) in Chl fluorescence is associated with the reduction of PQ, likely caused by the electrons accumulated in the stroma or cytosol during illumination transferred back (Asada et al., 1993; Mi et al., 1995; Shikanai et al., 1998), and therefore be used to evaluate the activity of cyclic electron flow (Deng et al., 2003). The PIR in WT became more apparent under the heat treatment, while the *ΔB* mutant did not show substantial PIR (**Figure 3E**) despite the heat treatment. The enhanced CEF in WT appeared synchronous to the thermal acclimation, suggesting the absence of the NDH-mediated CEF could be associated with the delay of acclimation to a higher temperature.

The maximum rate of Rubisco carboxylase activity (*V_c_*_max_), maximum rate of light-saturated photosynthetic electron transport (*J*_max_), and dark respiration rate were calculated from A-Ci curves (Sharkey et al., 2007) (**Table 1**; **Supplementary Figure 2**). The dark respiration rate of WT increased after heat treatment, but that of *ΔB* was not changed. *V_c_*_max_ at 25°C (*V_c_*_max25_) in WT slightly increased at 1 DAH, while *V_c_*_max25_ was not changed in *ΔB* after heating. Notably, the increased difference of *V_c_*_max_ at 35°C (*V_c_*_max35_) between WT and *ΔB* further indicated an inhibited Rubisco carboxylase activity in *ΔB* relative to WT. On the other hand, *J*_max_ at 35°C (*J*_max35_) were slightly higher in WT than in *ΔB* after heat treatment.

**Table 1:** A_n_, R_d_, V_cmax_, J_max_, and J_max_/V_cmax_ in WT and *ΔB* tobacco leaves.

	A_n_ (µmol m^-2^ s^-1^)	R_d_ (µmol m^-2^ s^-1^)	*V_cmax_* at 25°C (µmol m^-2^ s^-1^)	*V_cmax_* at 35°C (µmol m^-2^ s^-1^)	*J_max_* at 25°C (µmol m^-2^ s^-1^)	*J_max_* at 35°C (µmol m^-2^ s^-1^)	*J_max_* / *V_cmax_* at 25°C	*J_max_* / *V_cmax_* at 35°C
WT								
0DAH	11.92±1.36 b	1.11±0.20 b	41.10±2.92 ab		73.19±10.4 a		1.78±0.14 a	
1DAH	13.37±1.90 a	1.14±0.32 ab	44.91±2.80 a	105.60±6.58 a	56.31±6.64 b	100.03±11.8 ab	1.26±0.20 b	0.95±0.14 a
2DAH	13.66±1.62 a	1.40±1.24 a	43.22±8.26 ab	101.63±19.4 ab	56.63±4.14 b	100.61±7.34 a	1.32±0.12 b	0.99±0.12 ab
*ΔB*								
0DAH	10.61±0.78 c	1.14±0.42 b	40.63±5.80 ab		72.25±12.3 a		1.78±0.06 a	
1DAH	9.75±0.52 d	1.04±0.20 b	40.25±0.28 ab	94.65±0.68 b	53.72±2.10 b	95.44±3.74 ab	1.33±0.06 b	1.01±0.04 b
2DAH	12.38±1.84 b	0.91±0.18 b	39.28±4.58 b	92.35±10.8 b	53.33±1.28 b	94.76±2.30 b	1.36±0.16 c	1.03±0.12 b

A_n_, R_d_, V_cmax_, J_max_, and J_max_/V_cmax_ prior to (0 hour), 1 day, and 2 days after heating were measured and calculated according to the method of Sharkey et al. (2007). The response of photosynthetic assimilation rate (A_n_) to intracellular CO_2_ concentration (Ci) (A-Ci curve) measurements were performed at growth temperature under 600 µmol photons m^-2^ s^-1^ light intensity. The values with grey background were calculated values based on FvCB model. Values in the table are means SD (*n* = 4 plants), and different lower-case letters indicate significant (*P* < 0.05) differences assessed using two-way ANOVA.

### Distinct changes in transcript and protein levels between WT and NDH-B deficient mutant in response to heat treatment

**Figure 4:**
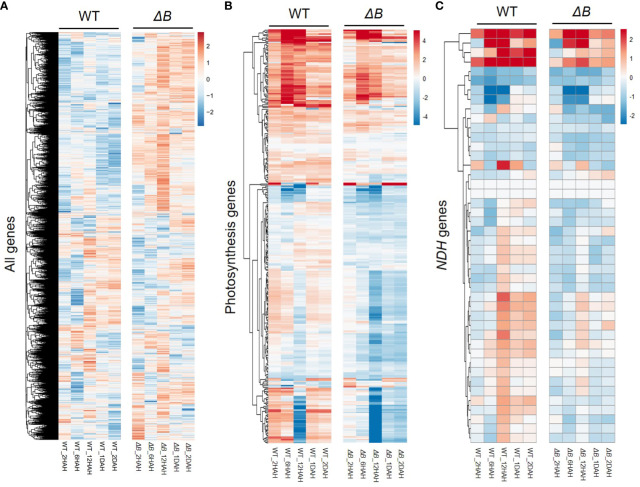
Distinct changes in transcript profiles between WT and *ndhB* mutant in response to heat treatment. Hierarchical clustering was shown for all the differentially expressed genes (DEGs) **(A)**, photosynthesis-related genes **(B)**, and nuclear-encoded NDH subunit genes **(C)** in WT and *ΔB* at each time points (2 hours, 6 hours, 12 hours, 1 day and 2 days after heating) relative to the gene expression before heat treatment. Fold changes over the time series are displayed on log2 scale: red = upregulated and blue = downregulated.

To assess the gene expression changes of WT and *ΔB* tobacco leaves in response to heat treatment, an RNAseq analysis was undertaken at different time points, in comparison with the expression levels before treatment. At the total transcriptome level, principal component analysis (PCA) showed clear gene expression variation following the time-points of heat treatment (**Supplementary Figure 3A**). Although the PCA indicates that the expression variation was more affected among timepoint rather than between WT and *ΔB*, the total expression heatmap showed that the expression patterns of *ΔB* mutant at each time-point were mostly distinct from those of WT (**Figure 4A**). At 2 HAH, there were 4644 differentially expressed genes (DEGs) in WT, including 2424 up-regulated and 2220 down-regulated genes; and there were 5409 DEGs in *ΔB* mutant, which included 2728 up-regulated and 2681 down-regulated genes. At 6 HAH and 12 HAH, there were more DEGs in both WT (12181 and 13031 DEGs) and *ΔB* (10821 DEGs and 11377 DEGs) (**Supplementary Figure 3B**). Considering the appearance of significant physiological inhibition at early stage of 2 HAH (**Figure 3C**), Gene ontology (GO) term enrichment analysis was undertaken, which revealed that up-regulated DEGs in WT at 2 HAH were enriched in multiple photosynthesis-related pathways, such as “light-harvesting”, “chloroplast organization”, “Photosystems” and “chloroplast thylakoid membrane” (**Supplementary Figure 4A**). The up-regulated DEGs at 2 HAH in *ΔB* were enriched in pathways that respond to stress, such as “response to heat”, “response to temperature stimulus”, “response to reactive oxygen species” and “response to abiotic stimulus”, instead of photosynthetic pathways (**Supplementary Figure 4C**). The hierarchal clustering of photosynthesis-related DEGs indicated that around two-thirds were up-regulated in WT at 2 HAH, 6 HAH and 24 HAH (1 DAH), whereas many of these genes were down-regulated in *ΔB* (**Figure 4B**). Further classification of photosynthetic genes (**Supplementary Figure 5**) showed that DEGs encoding PSII, PSI, and their light-harvesting proteins (Lhcbs and Lhcas) were most distinct between WT and *ΔB*.

**Figure 5:**
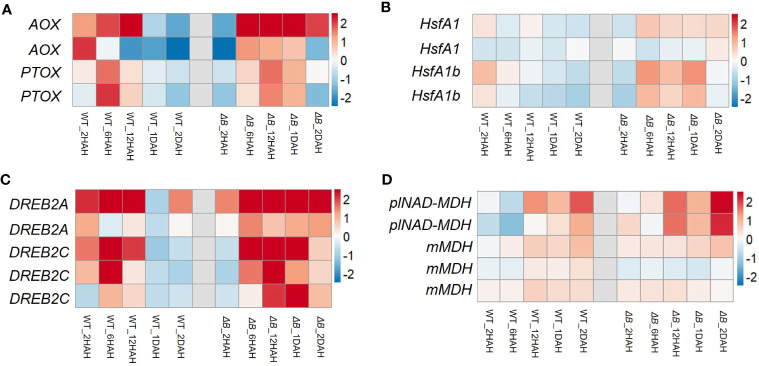
Heat induced gene expression changes of oxidases and regulators in WT and *ΔB*. Heat maps show the gene expression of alternative oxidase (AOX) and plastid terminal oxidase (PTOX) **(A)**, heat shock factors **(B)**, dehydration-responsive element-binding protein 2A (DREB2A), DREB2C **(C)**, plastidial NAD-dependent malate dehydrogenase (plNAD-MDH) and mitochondria malate dehydrogenase (mMDH) **(D)**, in WT and *ΔB* at each time points (2 hours, 6 hours, 1 day and 2 days after heating) relative to the gene expression before heat treatment. Fold changes over the time series are displayed on log2 scale: red = upregulated and blue = downregulated.

Around two-thirds of nuclear-encoded NDH subunit genes were up-regulated after heat treatment. Some of the up-regulations were temporary, that reached their peaks around 12 HAH, and subsequently returned toward previous levels (**Figure 4C**, **Supplementary Figure 5**). The temporary up-regulation was less observed in *ΔB* mutant. Another group of DEGs distinct between WT and *ΔB* were Rubisco-related genes (such as Rubisco small subunit gene and gene encoding Rubisco large subunit-binding protein), but not for Rubisco activase (**Supplementary Figure 5**). Apart from photosynthetic genes, RNA-seq also indicated the up-regulation of alternative oxidase (AOX) participating in respiration, plastoquinol terminal oxidase (PTOX), and some thermos-responsive transcription factors, such as class A1 heat shock factor (HsfA1) and dehydration-responsive element-binding protein 2A/2C (DREB2A/DREB2C). The up-regulation of these genes lasted longer in *ΔB* than in WT. The expression of genes encoding plastidial NAD-dependent malate dehydrogenase (plNAD-MDH) and mitochondrial malate dehydrogenase (mMDH) was also found increased in both *ΔB* and WT (**Figure 5**).

At the protein level, western blots indicated that the changes of photosystem proteins were different between WT and *ΔB* during the first 2 days of thermal acclimation. PsbA (D1) content of PSII tended to be increased in WT, but was transiently declined at 2 HAH in *ΔB*. PsaD of PSI was temporarily suppressed in WT, while it was in a downward trend in *ΔB*. Lhcb and Lhca were enhanced over the first 6 or 12 hours in WT but suppressed after 12 hours in *ΔB* (**Supplementary Figure 6B**). The protein abundances of NDH subunits in WT tobacco leaves showed temporary increases that correspond to gene expression pattern; ndhT, ndhS and ndhH from two different sub-complexes showed increased contents at the period from 2 HAH to 1 DAH, but returned to normal level at 2 DAH. Similar trends were found from other proteins contributing to CEF (PGR5/PGRL1) (**Supplementary Figures 6A, D**). In addition, a Blue-Native PAGE was undertaken, showing that the content of NDH-PSI supercomplex was increased at 1 DAH and 2 DAH compared to 0 DAH in WT, while it was absent in *ΔB* (**Supplementary Figure 6C**).

### Different accumulation of photosynthetic metabolites over day and night shift in WT and NDH-B deficient mutant under heat treatment

**Figure 6:**
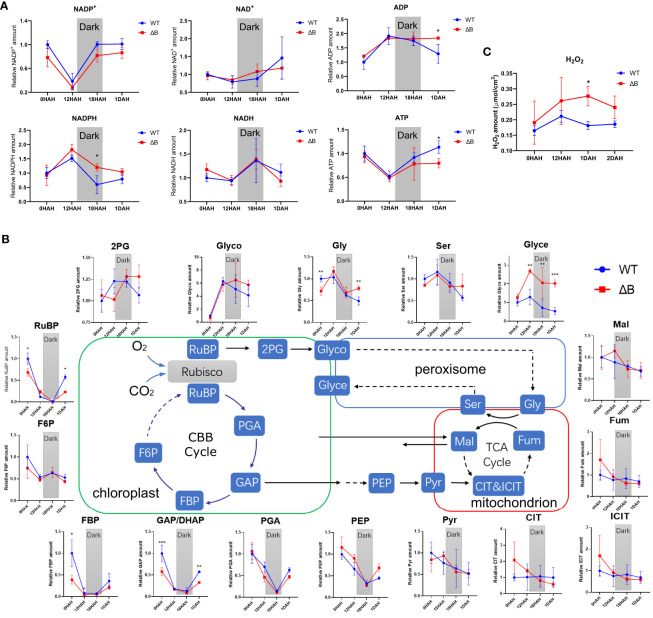
Different metabolic profiles in WT and *ndhB* mutant over day and night shift during early heat acclimation. **(A)** Line plots of NADP^+^, NADPH, NAD^+^, NADH, ADP and ATP amount changes in WT and *ΔB* plants over the first 24 hours of heat treatment. **(B)** Profile changes of the metabolites involved in Calvin–Benson–Bassham (CBB) cycle, photorespiration, and tricarboxylic acid (TCA) cycle in WT and *ΔB* plants over the first 24 hours of heat treatment. **(C)** Changes of hydrogen peroxide (H_2_O_2_) content in WT and *ΔB* plants among the 2 days of heat acclimation. The content of each photosynthetic metabolite was calculated by the peak area at their respective retention time (determined by mixed samples of standard metabolites) under reference MS parameters, and the peak area of each metabolite in WT at 0HAH was set unit “1.00” as control. Error bars represent standard deviation of four biological replicates. *P < 0.05, **P < 0.01, ***P < 0.001 according to two-way ANOVA.

In order to further dissect the mechanism behind the delayed photosynthetic thermal acclimation in *ΔB* mutant, we focused on the first day-night-day shift (including time points shortly before night and shortly before dawn) and performed a set of metabolism analysis. The two products of light reactions, NADPH and ATP, were used to drive CBB cycle. The NADPH amount of WT and *ΔB* increased at 12 HAH (before night) and reduced at 18 HAH (before dawn); its dehydrogenated product NADP^+^ had the opposite trend. However, *ΔB* accumulated a larger amount of NADPH than WT, and was not able to consume NADPH sufficiently during the night time (**Figure 6A**); that is, the mutant tended to accumulate more reducing power at the end of the diurnal cycle under increased temperature. ATP content decreased after heating, but recovered to higher level in WT at 1 DAH, while stayed at lower level in *ΔB* (**Figure 6A**). Contrary to NADPH, NADH was accumulated at 18 HAH, and its amount was similar in WT and *ΔB* within the first 24 hours under heating. To evaluate the effect of reducing power accumulation, the amount of hydrogen peroxide (H_2_O_2_) was analysed. As expected, H_2_O_2_ content increased at 12 HAH, and remained relatively higher level in *ΔB* than in WT (**Figure 6C**).

Apart from light reactions, majority of metabolites participating in the CBB cycle were markedly decreased upon temperature increase in both WT and *ΔB*. Ribulose bisphosphate (RuBP), the initial substrate of CBB cycle, decreased at 12 HAH and 18 HAH, and re-accumulated at 1 DAH but with significantly lower level in *ΔB* than in WT. The subsequent metabolites in CBB cycle, 3-phosphoglyceric acid (PGA), glyceraldehyde 3-phosphate/dihydroxyacetone phosphate (GAP/DHAP), and fructose-1,6 bisphosphatase (FBP), exhibited similar trends as RuBP; GAP, as the prime end-product of photosynthesis, was with lower amount in *ΔB* than in WT at 1 DAH (**Figure 6B**). At the end of the glycolysis process, phosphoenolpyruvate (PEP) exhibited similar decrease and increase pattern as CBB cycle metabolites, while the subsequent metabolites involved in respiration process, pyruvate (Pyr), citric acid (CIT), isocitric acid (ICIT), fumarate (Fum) and malate (Mal), did not show markable differences between WT and *ΔB* mutant. Unlike respiration, most of the metabolites participating in photorespiration were induced at 12 HAH and decreased overnight; and the amount of glyceric acid (Glyce) showed remarkably higher level in *ΔB* than in WT from 12HAH (**Figure 6B**).

## Discussion

This study investigated the responses of photosynthesis processes to increased environmental temperature in tobacco and the role of NDH-mediated cyclic electron transport in photosynthetic thermal acclimation. It was found that the WT improved its photosynthetic performance rapidly after exposure to moderate higher temperature, while the NDH-B deficient mutant acclimated clearly slower (**Figure 1**). Since missing one of the subunits could cause dysfunction of the whole NDH complex (Kofer et al., 1998; Shikanai et al., 1998; Rumeau et al., 2005), especially since the NDH-B subunit is located in the membrane arm (Shen et al., 2021), the delayed thermal acclimation of photosynthesis was not solely associated with the mutation of a single subunit, but was more likely related to the absence of NDH-mediated cyclic electron flow (CEF).

The contribution of NDH-mediated CEF to abiotic stress or acclimation is debated (Yamori et al., 2011; Suorsa et al., 2012; Essemine et al., 2016; Li et al., 2016). Suorsa et al. (2012) indicated that NDH complex did not contribute to photosynthetic acclimation to fluctuating light, based on a similar phenotype of Arabidopsis *ndho* mutant to WT under the condition. This observation has inspired us to further clarify whether or to what extent NDH complex may contribute to the acclimation process to mild heat stress, which is more commonly happened in the field than extreme heat conditions. As the transcript and protein regulation of thermal acclimation is rapid, which could be within 24 hr of temperature increase (Rashid et al., 2020), we considered using early and high temporal resolution for our measurements to identify the contribution of NDH-mediated CEF.

Earlier reports on cowpea showed that light reactions were induced under moderate heat stress, even though the total photosynthesis rate was inhibited (Osei-Bonsu et al., 2021). Interestingly, during thermal acclimation, our results demonstrate that the heat impacts on light reactions and CBB cycle appeared to be asynchronous in tobacco, especially that the inhibition of PSII activity in *ΔB* mutant recovered earlier than its carbon assimilation rate (**Figures 3A, B**). Unparallel acclimation of light reactions and CO_2_ assimilation could result in that reducing equivalents produced by light reactions could not be promptly consumed by CBB cycle, which was supported by increased accumulation of NADPH after heating, revealed by the metabolic results (**Figure 6A**). The hindered acclimation even in WT under full-light condition (**Figure 2**) is likely attributed to exacerbated redox imbalances enforced under 24-hour constant light.

To adapt to the increase in temperature, tobacco plants need to establish a new redox equilibrium in chloroplasts, which requires alternative pathways to divert excited electrons or consume excessive reducing power. The two main alternative electron flows in chloroplast are water-water cycle (WWC; Mehler-ascorbate peroxidase pathway) and CEF (Miyake, 2010). Increased H_2_O_2_ in *ΔB* suggested that greater portion of electrons might be flowing to WWC. This was supported by the up-regulated genes at 2HAH related to ROS and H_2_O_2_ responding pathways in heat treated *ΔB* (**Supplementary Figure 4**). As previous studies proposed (Casano et al., 2001; Lascano et al., 2003), the elevated content of H_2_O_2_ was responsible for the increased protein levels of NDH subunits in WT (**Supplementary Figure 6A**). The mechanism that H_2_O_2_ induces NDH-mediated CEF is advantageous in balancing ATP/NADPH ratio by redirecting electrons through CEF (Strand et al., 2015), but this mechanism is impeded in *ΔB*.

Except for being used in CBB cycle, chloroplast reducing power could be exported from the organelle via malate-oxaloacetate shuttle (Mal/OAA shuttle) (Raghavendra and Padmasree, 2003; Yoshida et al., 2006; Dietz et al., 2016; Dao et al., 2022). The gene expression of one of the key enzymes for Mal/OAA shuttle, plastidial NAD-dependent malate dehydrogenase (plNAD-MDH), were up-regulated (**Figure 5D**) (Zhao et al., 2020). Combined with the up-regulation of mitochondrial malate dehydrogenase (mMDH) genes, it is possible that part of the reducing equivalents were transferred from chloroplast to mitochondria through Mal/OAA shuttle. In addition, the Mal/OAA shuttle was proposed to be coupled with photorespiration at multiple levels (Dao et al., 2022). A recent study using fluorescent protein sensors to monitor dynamic changes of NADPH and NADH also indicated that photorespiration is the major NADH contributor in mitochondria (Lim et al., 2020). The levels of metabolites participating in photorespiration were induced after heating; particularly, the level of glyceric acid was remarkably accumulated in *ΔB* compared to the WT (**Figure 6B**). Considering that glyceric acid requires ATP in order to convert to 3-phosphoglycerate (3-PG), the accumulation of glyceric acid in *ΔB* might be also associated with the compromised production of ATP due to NDH deficiency (Betti et al., 2016; Walker et al., 2016). On the mitochondrial side, it is worth highlighting that the upregulation of *AOX* gene in *ΔB* persisted longer (**Figure 5**). The AOX pathway was generally thought to contribute to PSII photoprotection by dissipating excess reducing power from chloroplasts through the Mal/OAA shuttle (Raghavendra and Padmasree, 2003; Yoshida et al., 2006; Dinakar et al., 2010). Besides, it was reported to contribute in maintaining photorespiration, which alleviates over-reduction in the chloroplasts as well (Zhang et al., 2017; Li et al., 2020).

Apart from the above alternative pathways, PGRL5/PGRL1-mediated CEF was also responding to heat treatment, as the protein contents of PGRL5/PGRL1 increased in WT during the process (**Supplementary Figures 6A, D**). The PGRL5/PGRL1-CEF was considered to be the major CEF in C3 plants (Munekage et al., 2002; Munekage et al., 2004; Ma et al., 2021). Severe phenotypes were observed in the *pgr5* mutant under non-extreme stress (Suorsa et al., 2012). However, under moderate heat stress, without the obvious phenotype in *ndh* mutants, the effects of NDH-CEF was easy to be ignored. With this work, we are convinced that NDH-CEF was induced to participate in rapid acclimation to moderate temperature increase, and the acclimation process was also dependent on the night period. Under global warming, the ability to maintain redox status and quickly adapt to temperature changes is beneficial to plant growth and final yield (Challinor et al., 2014). The known CEF pathways together might have contributed to the rapid adaptation to increasing day and night temperatures, and thus the regulation of CEF is worth to be further studied, as potential engineering targets to improve crop heat resistance.

## Materials and methods

### Plant material and temperature treatments

Wild type tobacco (*Nicotiana tabacum cv Xanthi*), *ΔB* mutants in which the chloroplastic *NDHB* gene was inactivated (Shikanai et al., 1998) and *ΔCJK* mutant in which the chloroplastic *NDHC, NDHJ* and *NDHK* genes were inactivated (Takabayashi et al., 2002) were cultivated in a phytotron (about 500–600 µmol photons m^-2^ s^-1^, 16 hours light at 25 °C and 8 hours dark at 20 °C, 65% relative humidity). Six weeks old tobacco plants were transferred into growth cabinets with independent temperature control for heat treatment, in which temperature was set to 35 °C day and 30 °C night (with a control cabinet set to 25 °C day and 20 °C night). For every batch of tobacco plants used for experiment, at least 18 pots each for WT and *ndh* mutants were planted, which ensures that there are 3-4 replicates for measurement or sampling at each time point. For heat treatment under continuous light, temperature was set to 35 °C all day.

### Gas exchange measurements

Gas exchange was measured on mature tobacco leaves (third leaf from the top) prior to and after heat treatment. LI-6800 instrument equipped with 2 cm^2^ cuvettes and a 6800-01A multiphase flash fluorometer (a combination light source and chamber for gas exchange measurement) (Li-COR, Lincoln, NE, USA) was used to measure the net photosynthetic CO_2_ assimilation rates (A_n_). A_n_ was measured with the following settings: 600 (for temperature-response curve) or 1000 (for light-saturated An) µmol photons m^-2^ s^-1^, 400 μmol mol^-1^ CO_2_, flow rate of 500 μmol s^-1^, and relative humidity of 65%. All photosynthesis measurements were taken at least 2 hours after the growth cabinet lights were turned on.

During the measurements of the temperature-response curves, the measuring chamber temperature was heated at 1 °C per minute from 20 °C to 45 °C with the LI-6800 auto-control program, and An was recorded at 30-s intervals. The x-axis of temperature-response curve was leaf temperature, and the y-axis value corresponding to the vertex were taken as the maximum A_n_.

The A-Ci curve measurements were performed using the auto program settings of LI-6400 instrument with the following sequence of CO_2_ concentrations: 400, 200, 50, 100, 150, 300, 400, 500, 600, 800, 1000, 1200 μmol mol^-1^ CO_2_ (**Supplementary Figure 3**). CO_2_ levels were changed at 1-2 min intervals. A-Ci analyses were performed and related photosynthetic parameters were calculated according to Sharkey et al. (2007).

### Chlorophyll fluorescence measurements

Chlorophyll fluorescence parameters were measured using a portable modulated chlorophyll fluorometer, PAM-2000 (Walz, Effeltrich, Germany). Slow kinetics curve was measured on dark-adapted tobacco plants (pre-dark-treatment for at least 2 hours), started with a light-saturated pulse, application of actinic light (970 µmol m^-2^ s^-1^) after 40 second interval, and then light-saturated pulses every 20 seconds until fluorescence reached steady state. Chlorophyll fluorescence parameters were calculated as previous studies described (Bjorkman and Demmig, 1987; Krall and Edwards, 1992; Schultz, 1996; Kramer et al., 2004). The parameters included maximum photochemical efficiency (F_v_/F_m_), quantum yield of Photosystem II (φ_II_), and non-photochemical quenching (NPQ).

Post-illumination rise (PIR) of chlorophyll fluorescence was measured as previous studies described (Asada et al., 1993; Mi et al., 1995; Shikanai et al., 1998). PIR is the transient increase of chlorophyll fluorescence after actinic light turned off indicating re-reduction of the quinone pool in the dark, which is associated with the activity of NDH and correlates with the changes in NDH mediated CEF. The measurement with PAM-2000 was performed with an actinic light of 600 µmol m^-2^ s^-1^, illuminated until fluorescence reached steady-state, and the fluorescence curve was recorded for further 2 minutes after shut down of the actinic light.

### RNAseq and transcriptome data analysis

Mature leaves of tobacco (third leaf from the top) were harvested prior to and after the temperature treatment, immediately frozen in liquid N_2_ and stored at -80 °C. RNA samples were extracted from the leaf tissue using TRIzol® Reagent (Invitrogen, Massachusetts, USA) and genomic DNA was removed using DNase I (TaKara, Shiga, Japan). The RNA quality was determined by 2100 Bioanalyser (Agilent Technologies, CA, USA) and quantified using ND-2000 (NanoDrop Technologies, Delaware, USA).

Transcriptome library for RNAseq was prepared from 1 μg of total RNA for each sample, using TruSeqTM RNA sample preparation Kit from Illumina® (San Diego, CA, USA). After sequencing, the raw paired-end reads were trimmed and quality controlled by SeqPrep (https://github.com/jstjohn/SeqPrep) and Sickle (https://github.com/najoshi/sickle) with default parameters. The clean reads were aligned to *Nicotiana tabacum* reference genome (GCF_000715135.1, https://www.ncbi.nlm.nih.gov/genome/425?genome_assembly_id=274804) using HISAT2 (http://ccb.jhu.edu/software/hisat2/index.shtml) software (Kim et al., 2015), and reads were mapped assembled by StringTie (https://ccb.jhu.edu/software/stringtie/index.shtml?t=example) in a reference-based approach (Pertea et al., 2015). DEGs were identified according to the expression level of each transcript based on transcripts per million reads (TPM). Differential expression analysis was performed by DESeq2 (Love et al., 2014)/DEGseq (Wang et al., 2010)/EdgeR (Robinson et al., 2010); and if |log2FC| > 1 and Q value ≤ 0.05 (DESeq2 or EdgeR) or Q value ≤ 0.001 (DEGseq), the gene was identified as a DEG. Furthermore, functional-enrichment analysis including GO and KEGG were undertaken to identify which biological and metabolic pathways that DEGs were significantly enriched in. The statistical tests used were Fisher’s test (significance level of P < 0.05) and Benjamini-Yekutieli procedure (FDR < 0.05).

### Protein abundance assessment

Total leaf protein was extracted using a protein extraction buffer containing 50 mM Tris-HCl (pH 8.0), 0.25 M sucrose, 2 mM dithiothreitol (DTT), 2 mM EDTA, and 1 mM phenylmethylsulfonyl fluoride (PMSF). Protein content was evaluated by western blot, where the extracted proteins (>20 μg for each sample loading) were separated by SDS-PAGE (12.5% SDS-PAGE gels) and transferred onto a PVDF membrane. After blocking the membrane with 5% w/v skim-milk in TBST (150 mM NaCl, 20 mM Tris, 0.1% Tween, pH 7.6) for 1 hour, it was incubated in a primary antibody solution (1:5000 primary antibody, 5% w/v skim-milk powder in TBST) for 1 hour. The membrane was then washed with TBST for 1 hour and incubated with a secondary antibody (1% w/v skim-milk powder in TBST) for an additional 1 hour. The membrane was finally visualized using ECL Plus reagent (Epizyme, SQ201). The specific antibodies used for detecting photosynthetic proteins were obtained from Orizymes Biotechnologies Company (Shanghai), including NDH subunits, PGR5/PGRL1, PsaD, PsbA, Lhca1, Lhcb1 and Actin.

BN-PAGE gel was prepared according to Jarvi et al. (2011). Tobacco leaves were sampled and homogenized in STN medium at 4 °C (0.4 M sucrose, 50 mM Tris-HCl pH 7.6, 10 mM NaCl). After low-speed (200 g for 3 min) and high-speed (5000 g for 10 min) centrifugation at 4°C, the chloroplasts collected were ruptured by TN medium (50 mM Tris–HCl pH 7.6, 10 mM NaCl). The thylakoid membranes were collected by centrifugation at 8000 g for 5 min at 4°C, and suspended in solubilization buffer [25 mM BisTris-HCl, pH 7.0, 10 mM MgCl2, 20% (v/v) glycerol]. Thylakoid membranes containing 0.5 mg ml^−1^ chlorophyll were solubilized with 2% (w/v) n-dodecyl-β-maltoside (DDM) by gentle agitation on ice for 1 hour. The samples were separated by Native-PAGE undertaken at 4 °C by increasing the voltage gradually from 50V to 200 V during the 5.5 h run. After running, the gel was stained with a coomassie blue solution (0.1% Coomassie Blue R250 in 10% aceticacid, 40% methanol and 50% H_2_O) for 1 hour and de-stained with a de-staining solution (10% acetic acid, 40% methanol and 50% H2O) for 12 hr. Band identifications are taken based on previous study (Jarvi et al., 2011).

### Metabolite analysis of leaf tissue

Tobacco leaf discs (1-cm-diameter) were collected prior to and after heat treatment. The treatment was started at 9 am, and subsequent sampling was performed at 9 pm (12 hours, 2 hours before dark), 3 am (18 hours, 4 hours after dark), and 9 am (1DAH). At each time point 3 tissue replicates from WT and *ΔB* were sampled, immediately frozen in liquid nitrogen, and ground to powder with steel balls at later stage. To extract metabolites, 4°C pre-cooled methanol: chloroform (7:3, v/v) was added to the samples. The samples were then incubated at -20°C for 3-4 hours, and 560 μL cold deionized water was added to each sample. After centrifugation at 2200 g (4°C), the supernatant was transferred to new tubes. A 50% (v/v) methanol solution was mixed with the precipitation, centrifuged again, and the supernatant was transferred to the previous tubes. Extracts were collected and stored at -80°C after being filtered by an organic phase filter. For sample loading, the metabolite extract was injected into a QTRAP 6500+ system (Sciex, Danaher Corporation, USA). Separation was conducted using a Luna® NH_2_ LC column (3 µm, 100 × 2 mm, Phenomenex, California, USA), with gradients from 20% (v/v) acetonitrile solution to solvent A (20mM ammonium acetate in 5% acetonitrile solution, pH 9.5), and to solvent B (acetonitrile). The elution started with different solvent percentages for different durations and ended with 85% solvent B for 3 minutes (0~1 min, 15% A and 85% B; 1~8 min, 70% A and 30% B; 8~22 min, 95% A and 5% B; 22~25 min, 15% A and 85% B). The column eluent was connected to the mass spectrometer fitted with an electrospray. The detection mode was set to negative ion mode, and the scan mode was set to multi reaction monitoring (MRM). For each sample, molecular features were defined based on retention time and mass (m/z) with Analyst® software 1.6.3 (Sciex, Danaher Corporation, USA). The carbon-metabolism metabolites as well as cofactors NAD(P)H、NAD(P)^+^、ADP and ATP were determined according to the chromatographic peaks calculated by the software, and the relative amounts of metabolites among different time-points were calculated by the chromatographic peak areas.

## Statistical analysis

For all measurements and samplings before or after heat treatment, three to four replicates from separate tobacco plants were chosen. Two-way ANOVA was performed on photosynthetic gas-exchange and chlorophyll fluorescence experiments comparing among WT and DB at different timepoints. These statistical analysis was performed using GraphPad Prism (v8) software.

## Data availability statement

The datasets presented in this study can be found in online repositories. The names of the repository/repositories and accession number(s) can be found below: RNA-seq data are available under the BioProject identifier PRJNA961907.

## Author contributions

YZ: Conceptualization, Data curation, Investigation, Validation, Writing – original draft. YF: Investigation. XL: Data curation. XZ: Investigation. QZ: Investigation. PW: Conceptualization, Funding acquisition, Project administration, Resources, Supervision, Writing – review & editing.

## Supplementary material

Supplementary Figure 1 Delayed photosynthetic heat acclimation in *ndhCJK* mutant over day and night shift. **(A)** Temperature-response curves of relative A_n_ at different time points after heating in *ΔCJK*. **(B)** Line plots of T_max_ changes of WT and *ΔCJK* plants after temperature shifting. Values represent the differences between the T_max_ at corresponding time points and the T_max_ before heating. **(C)** Relative A_n_ at 40°C at different time points after heating. A_n_ was measured under 600 µmol photons m^-2^ s^-1^, 400 µmol mol^-1^ CO_2_, and from 25°C to 40°C increasing temperature **(A)** or at 40°C **(C)**. Relative A_n_ values were normalized to the maximum values of A_n_. Shadow area **(A)** or error bars **(C)** represent standard deviation of four biological replicates. Different lower-case letters indicate significant (*P* < 0.05) differences by two-way ANOVA **(C)**.

Supplementary Figure 2 The response of photosynthetic assimilation rate (A_n_) to intracellular CO_2_ concentration (Ci) (A-Ci curves) measured at growth temperature under 600 µmol photons m^-2^ s^-1^ light intensity in WT and *ΔB* tobacco prior to (Day 0), 1 day, and 2 days after heating. **(A)** A-Ci curves of WT and *ΔB* at 25°C; **(B)** A-Ci curves of WT and *ΔB* 1 day after heating at 35°C; **(C)** A-Ci curves of WT and *ΔB* 2 days after heating at 35°C. Shadow area represents standard deviation of four biological replicates.

Supplementary Figure 3 Principal component analysis (PCA) of normalized gene expression values for each sample and Venn diagram of differentially expressed genes. **(A)** PCA analysis of samples prior to (0 hour), 2 hours, 6 hours, 12 hours, 1 day, and 2 days after heat treatment. **(B–E)** Overlap of the differentially expressed genes (DEGs) at 2, 6, and 12 HAH.

Supplementary Figure 4 Gene ontology (GO) term enrichment among differentially expressed genes (DEGs) at 2 HAH in WT and *ΔB*. **(A–D)** show the GO term enrichment among up-regulated DEGs in WT **(A)**, down-regulated DEGs in WT **(B)**, up-regulated DEGs in *ΔB* **(C)**, and down-regulated DEGs in *ΔB* **(D)**. The enriched photosynthetic pathways were circled with red box, and the enriched stress-response pathways were circled with blue box.

Supplementary Figure 5 Transcript profiles of photosynthetic genes in WT and *ΔB* during heat treatment. Hierarchal clustering of the differentially expressed genes (DEGs) grouped by different photosynthetic components was shown in WT and *ΔB*, at each time points (2 hours, 6 hours, 1 day and 2 days after heating) relative to the gene expression before heat treatment. Fold changes over the time series are displayed on log2 scale: red = upregulated and blue = downregulated.

Supplementary Figure 6 The contents of photosystem proteins and NDH complexes in WT and *ΔB* during heat treatment. **(A)** Western blot assay of NDH subunit and PGR5/PGRL1 proteins in WT leaves sampled prior to (0 hour), 2 hours, 6 hours, 12 hours, 1 day, and 2 days after heating. **(B)** Western blot assay of photosystem proteins PsbA, PsaD, Lhcb1 and Lhca1 in WT leaves sampled prior to (0 hour), 2 hours, 6 hours, 12 hours, 1 day, and 2 days after heating. **(C)** Blue native polyacrylamide gel electrophoresis (BN-PAGE) analysis of isolated thylakoid membranes from WT and *ΔB* prior to (0 hour), 1 day, and 2 days after heating. Band identifications are referenced from Jarvi et al. (2011). NDH-PSI super-complex bands were circled with red box. **(D)** Western blot assay of PGRL1 protein in *ΔB* leaves sampled prior to (0 hour), 2 hours, 6 hours, 12 hours, 1 day, and 2 days after heating. Actin was used as an internal control.
