# Supplementary figures and images for: Deficiency in NDH-cyclic electron transport retards heat acclimation of photosynthesis in tobacco over day and night shift
Source: Front Plant Sci. 2023 Oct 31;14:1267191. doi: 10.3389/fpls.2023.1267191 (PMC10644794; doi:10.3389/fpls.2023.1267191)

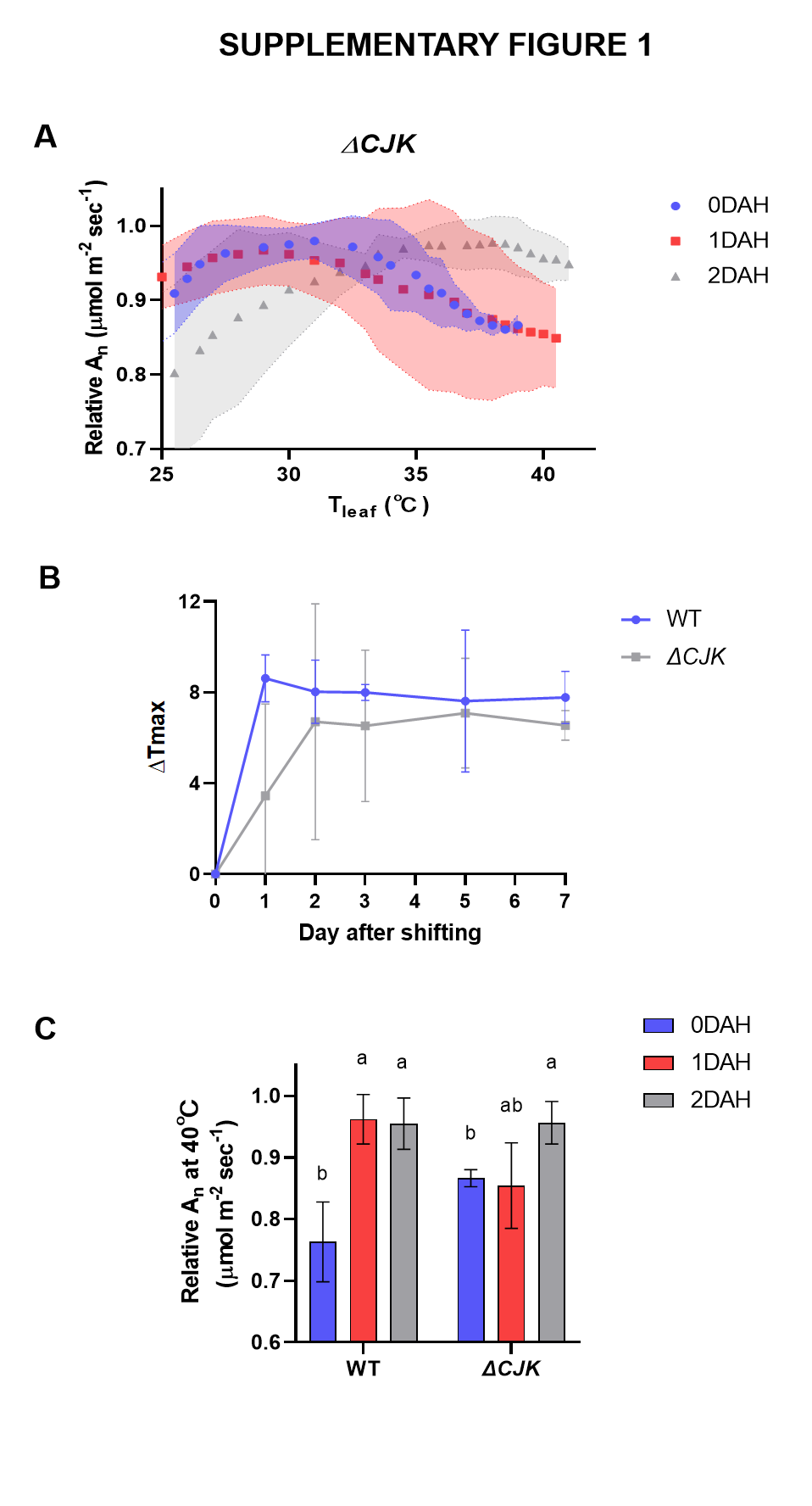

Supplement: Supplementary Figure 1 — Delayed photosynthetic heat acclimation in ndhCJK mutant over day and night shift. (A) Temperature-response curves of relative An at different time points after heating in ΔCJK. (B) Line plots of Tmax changes of WT and ΔCJK plants after temperature shifting. Values represent the differences between the Tmax at corresponding time points and the Tmax before heating. (C) Relative An at 40°C at different time points after heating. An was measured under 600 µmol photons m-2 s-1, 400 µmol mol-1 CO2, and from 25°C to 40°C increasing temperature (A) or at 40°C (C). Relative An values were normalized to the maximum values of An. Shadow area (A) or error bars (C) represent standard deviation of four biological replicates. Different lower-case letters indicate significant (P < 0.05) differences by two-way ANOVA (C). [file Image_1.png]

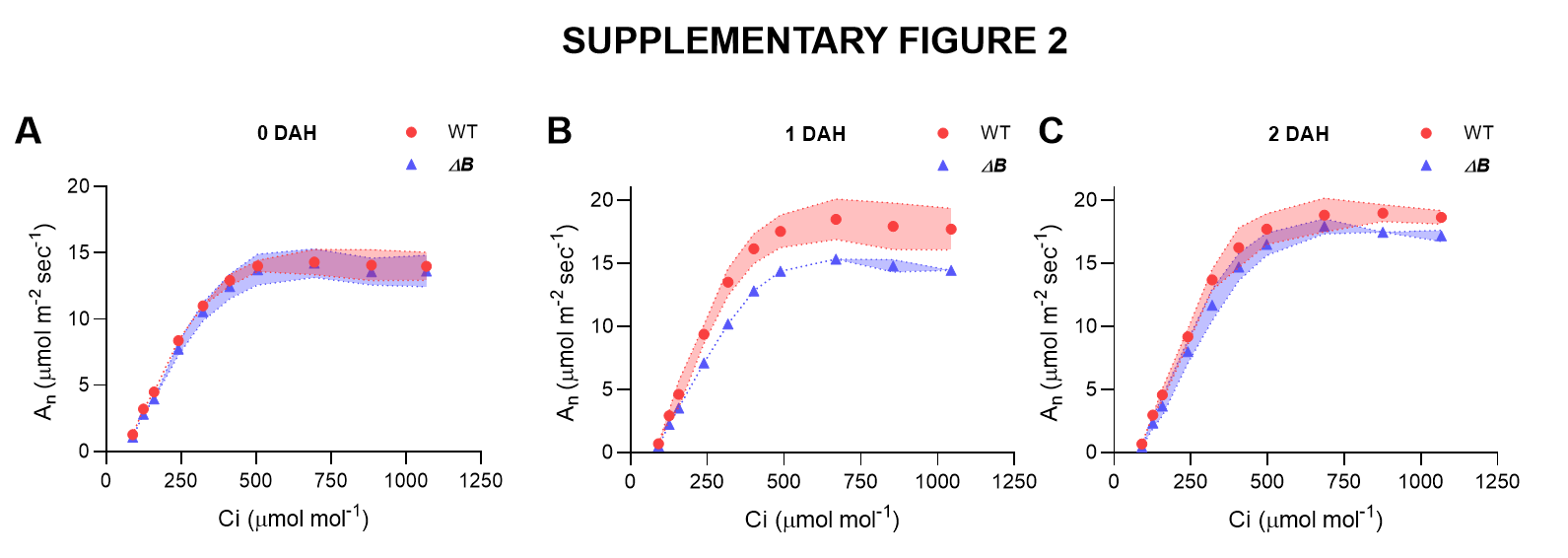

Supplement: Supplementary Figure 2 — The response of photosynthetic assimilation rate (An) to intracellular CO2 concentration (Ci) (A-Ci curves) measured at growth temperature under 600 µmol photons m-2 s-1 light intensity in WT and ΔB tobacco prior to (Day 0), 1 day, and 2 days after heating. (A) A-Ci curves of WT and ΔB at 25°C; (B) A-Ci curves of WT and ΔB 1 day after heating at 35°C; (C) A-Ci curves of WT and ΔB 2 days after heating at 35°C. Shadow area represents standard deviation of four biological replicates. [file Image_2.png]

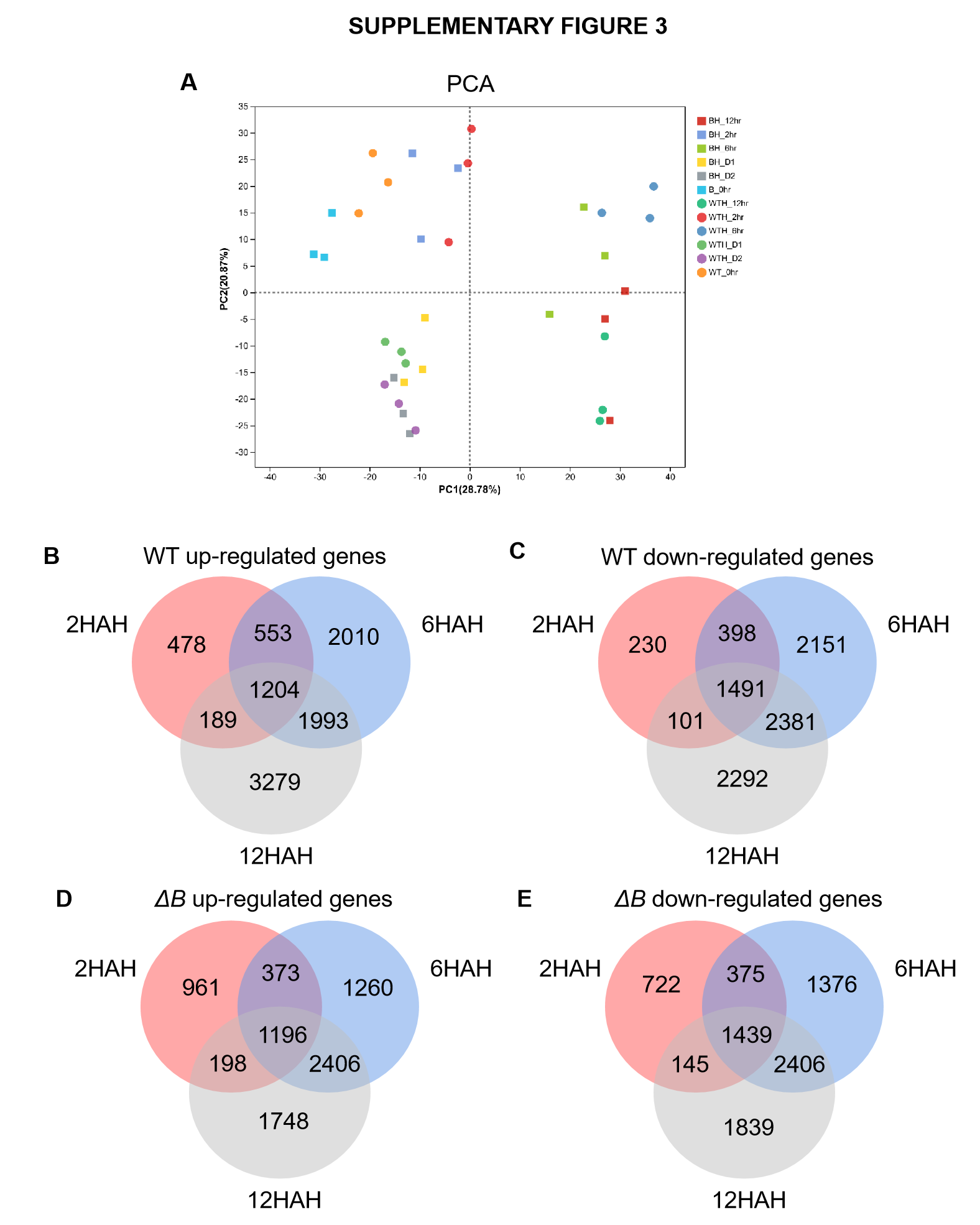

Supplement: Supplementary Figure 3 — Principal component analysis (PCA) of normalized gene expression values for each sample and Venn diagram of differentially expressed genes. (A) PCA analysis of samples prior to (0 hour), 2 hours, 6 hours, 12 hours, 1 day, and 2 days after heat treatment. (B–E) Overlap of the differentially expressed genes (DEGs) at 2, 6, and 12 HAH. [file Image_3.png]

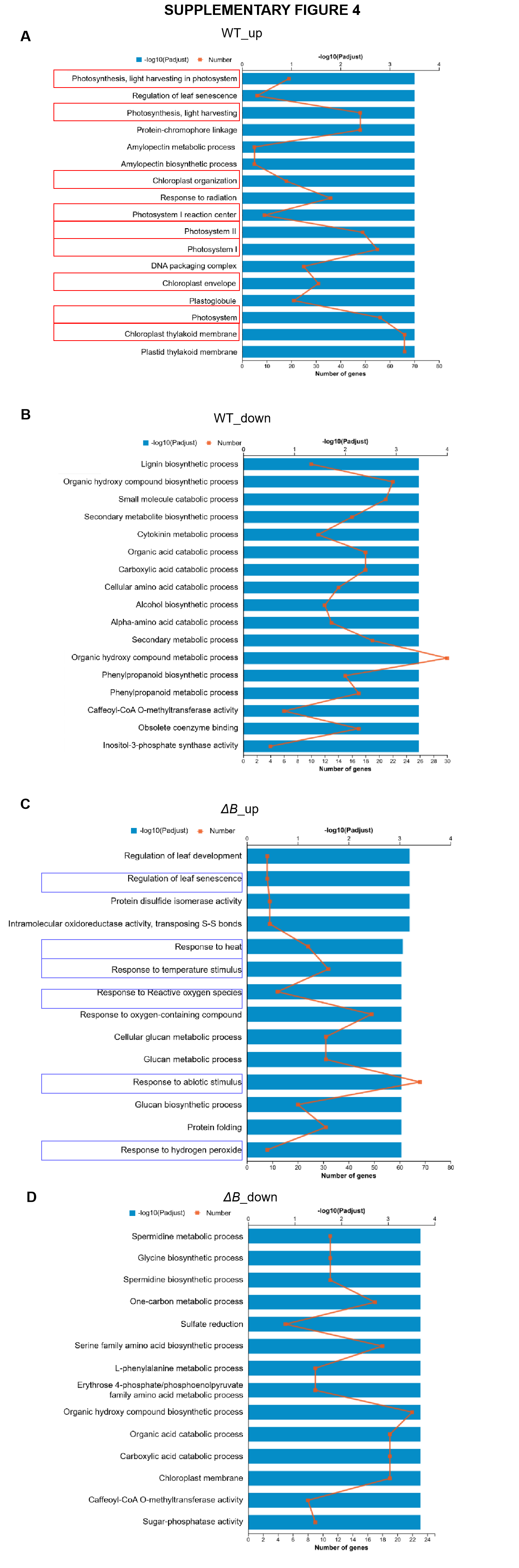

Supplement: Supplementary Figure 4 — Gene ontology (GO) term enrichment among differentially expressed genes (DEGs) at 2 HAH in WT and ΔB. (A–D) show the GO term enrichment among up-regulated DEGs in WT (A), down-regulated DEGs in WT (B), up-regulated DEGs in ΔB (C), and down-regulated DEGs in ΔB (D). The enriched photosynthetic pathways were circled with red box, and the enriched stress-response pathways were circled with blue box. [file Image_4.png]

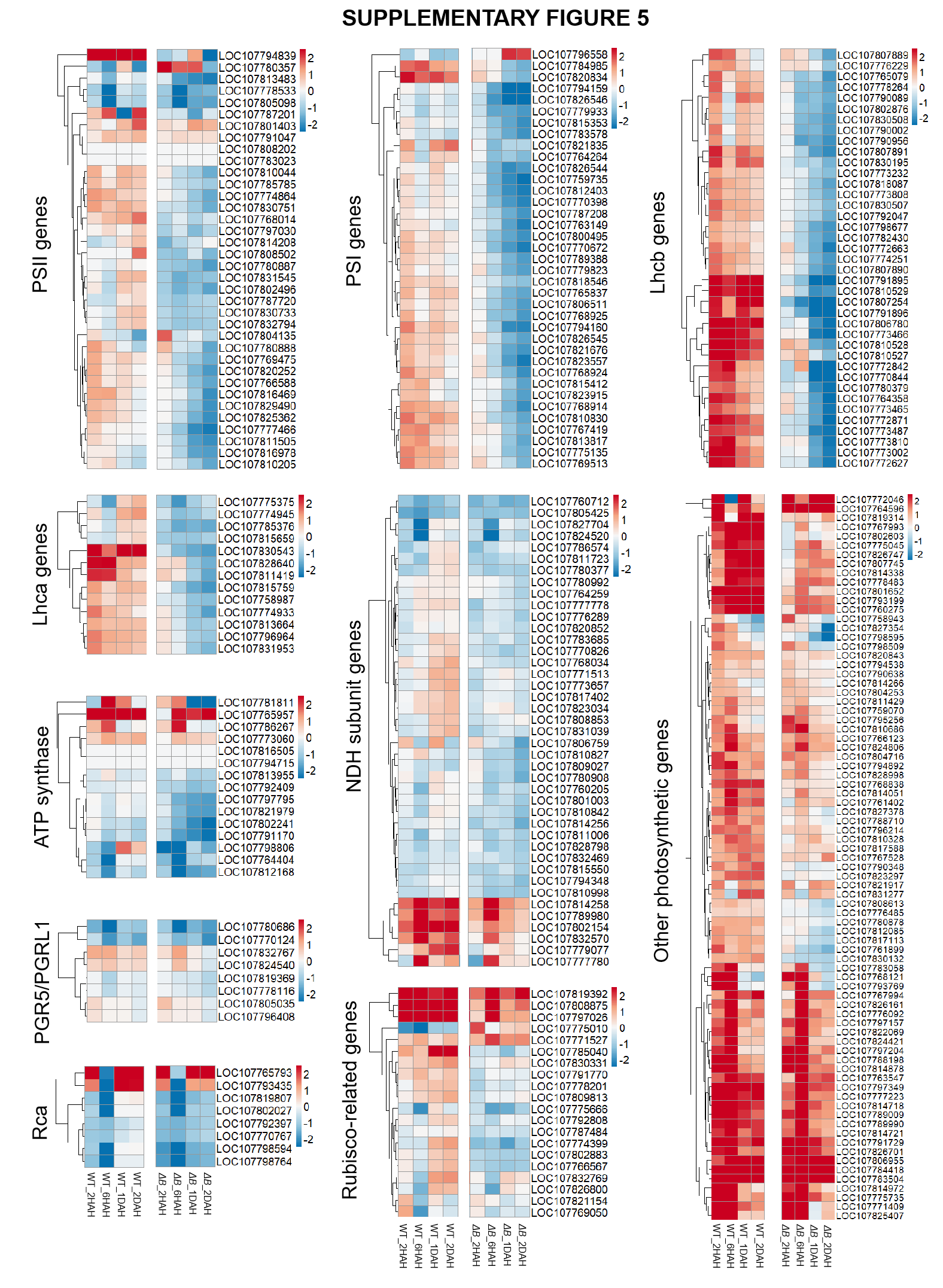

Supplement: Supplementary Figure 5 — Transcript profiles of photosynthetic genes in WT and ΔB during heat treatment. Hierarchal clustering of the differentially expressed genes (DEGs) grouped by different photosynthetic components was shown in WT and ΔB, at each time points (2 hours, 6 hours, 1 day and 2 days after heating) relative to the gene expression before heat treatment. Fold changes over the time series are displayed on log2 scale: red = upregulated and blue = downregulated. [file Image_5.png]

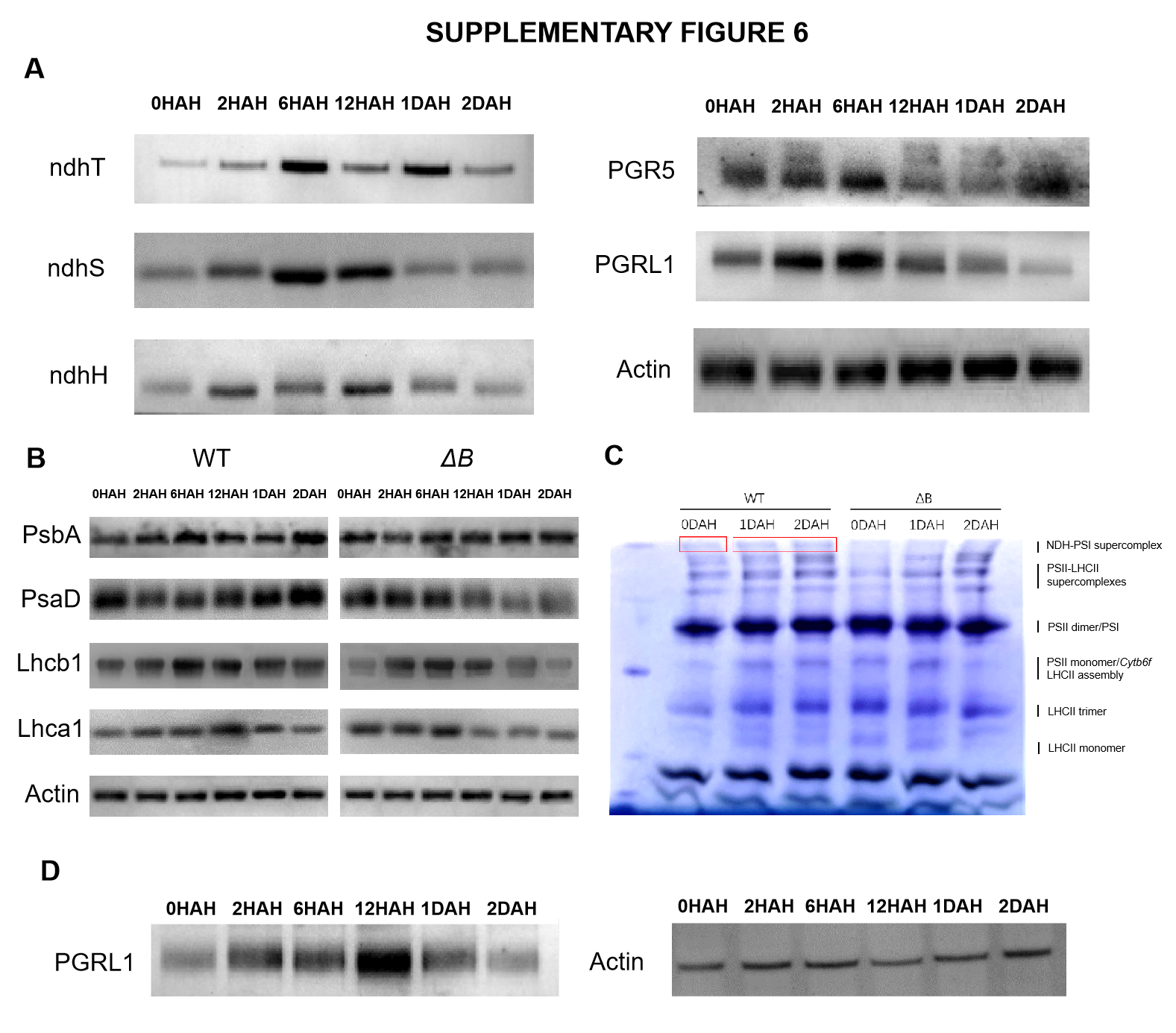

Supplement: Supplementary Figure 6 — The contents of photosystem proteins and NDH complexes in WT and ΔB during heat treatment. (A) Western blot assay of NDH subunit and PGR5/PGRL1 proteins in WT leaves sampled prior to (0 hour), 2 hours, 6 hours, 12 hours, 1 day, and 2 days after heating. (B) Western blot assay of photosystem proteins PsbA, PsaD, Lhcb1 and Lhca1 in WT leaves sampled prior to (0 hour), 2 hours, 6 hours, 12 hours, 1 day, and 2 days after heating. (C) Blue native polyacrylamide gel electrophoresis (BN-PAGE) analysis of isolated thylakoid membranes from WT and ΔB prior to (0 hour), 1 day, and 2 days after heating. Band identifications are referenced from Jarvi et al. (2011). NDH-PSI super-complex bands were circled with red box. (D) Western blot assay of PGRL1 protein in ΔB leaves sampled prior to (0 hour), 2 hours, 6 hours, 12 hours, 1 day, and 2 days after heating. Actin was used as an internal control. [file Image_6.png]

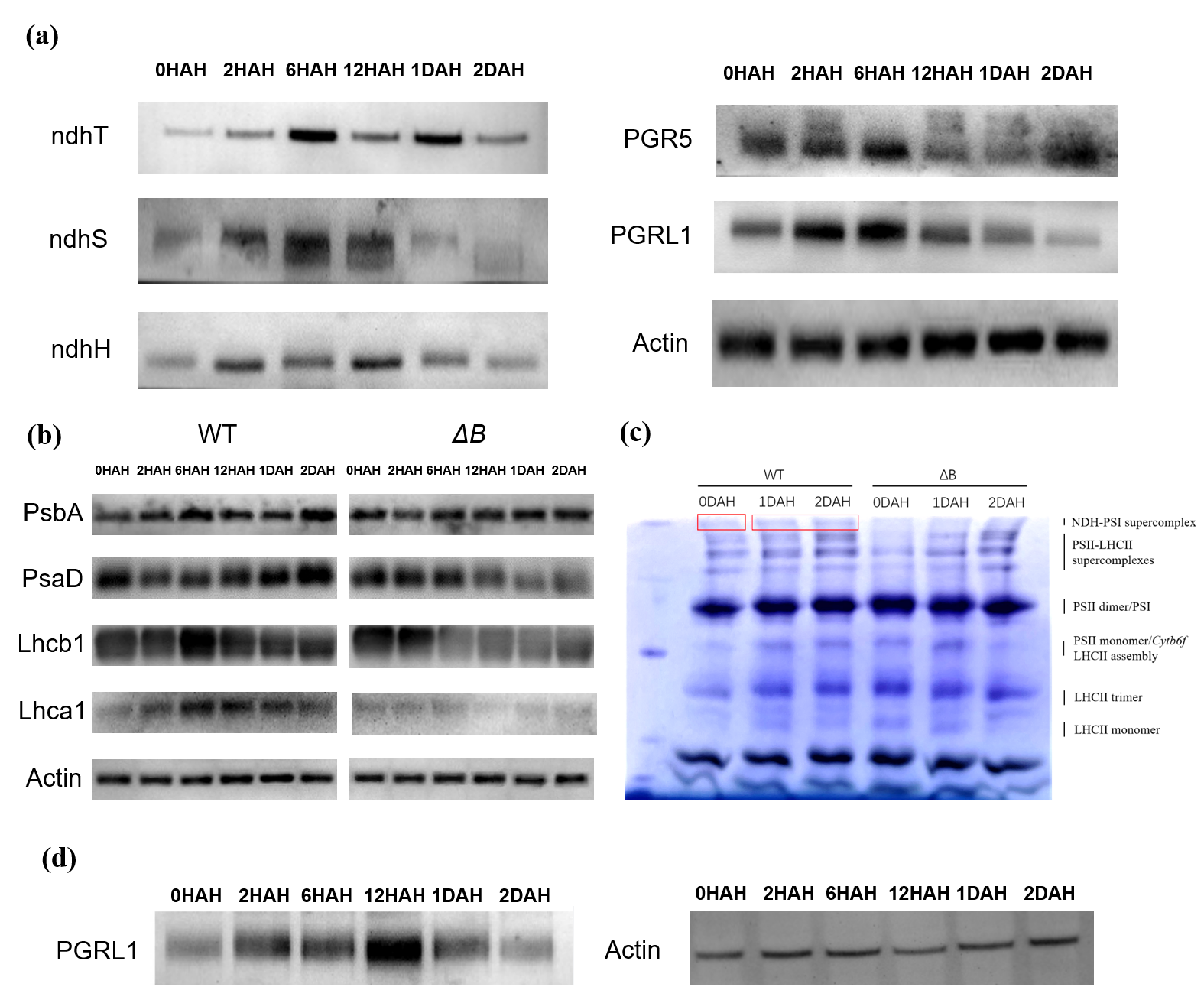

Supplement: Supplementary file 7 [file Image_7.png]
